# Psychometric validation of the Chinese version of the ethical decision-making competence scale in mainland Chinese nursing students

**DOI:** 10.3389/fmed.2026.1749960

**Published:** 2026-05-15

**Authors:** Xixi Li, Wei Mi, Qing Yang, Xiaoli Quan, Fuyan Tan, Lu Wang, Yu Li, Keyang Zhou

**Affiliations:** 1 Health Management Center, Suining Central Hospital, Suining, China; 2 Midwifery Teaching and Research Office, School of Nursing, Hunan University of Medicine, Huaihua, China; 3 Nursing Department, Nursing Research Office, Hunan University of Medicine General Hospital, Huaihua, China; 4 Department of Obstetrics, Hunan University of Medicine General Hospital, Huaihua, China; 5 Smart Health and Aging Research and Teaching Office, Luoyang Polytechnic, Luoyang, China; 6 Department of Internal Medicine Nursing, School of Nursing, Guizhou University of Medicine, Guiyang, China; 7 Teaching and Research Office of Obstetrics and Gynaecology Nursing, Zunyi Medical and Pharmaceutical College, Zunyi, China

**Keywords:** ethical decision-making, factor analysis, nursing students, reliability, validity

## Abstract

**Introduction:**

With the rapid transformation of global healthcare systems, ethical decision-making competence has become a critical component of nursing education. However, in mainland China, the assessment of nursing ethics remains underdeveloped, and validated instruments applicable across diverse educational levels are limited.

**Methods:**

A total of 1,407 nursing students from four provinces in China were recruited. The Ethical Decision-Making Competence Scale (EDM-CS) was translated and culturally adapted through forward translation, synthesis, back-translation, expert review, and pilot testing. Reliability was assessed using Cronbach’s alpha, split-half reliability, and test–retest reliability. Validity was examined through content validity indices, exploratory factor analysis (EFA), confirmatory factor analysis (CFA), convergent validity, and Heterotrait-Monotrait (HTMT) ratios.

**Results:**

The Chinese EDM-CS retained the original four-factor structure. The overall Cronbach’s alpha coefficient was 0.946, with subscale coefficients ranging from 0.869 to 0.904. Split-half reliability was 0.933 and test–retest reliability was 0.856. The item-level content validity index ranged from 0.833 to 1.000, and the scale-level content validity index was 0.920. EFA extracted four factors explaining 71.07% of the total variance, with factor loadings ranging from 0.730 to 0.846 and no salient cross-loadings. AVE values ranged from 0.518 to 0.711, CR values ranged from 0.870 to 0.907, and HTMT ratios ranged from 0.486 to 0.818.

**Conclusion:**

The Chinese version of the EDM-CS demonstrates robust psychometric properties and provides a standardized, theoretically grounded instrument for assessing ethical decision-making competence among nursing students in mainland China. The findings support the cross-cultural applicability of Rest’s Four-Component Model and provide empirical support for nursing ethics education and evaluation in mainland China.

## Introduction

Amid the ongoing transformation of global healthcare systems, nursing has become an essential component of modern medical services (1). The quality of nursing care directly influences patient outcomes and the overall healthcare experience. Ethical competence is a core dimension of care quality because it helps safeguard patients’ rights, sustain therapeutic nurse–patient relationships, and support high-quality healthcare delivery (2).

In recent years, China has made substantial progress in medical technology, with continuous advances in diagnostic and therapeutic approaches. By contrast, comparatively limited attention has been paid to the development and assessment of nursing ethical competence in mainland China (3). Current nursing ethics education remains insufficiently systematic and targeted, and well-validated, locally applicable instruments are still lacking (4, 5). This gap restricts the implementation of targeted educational interventions and may ultimately affect the quality of nursing practice (6).

China has established one of the largest medical education systems in the world, and nursing education follows a multi-level structure that includes initial, continuing, and postgraduate education. Initial education comprises vocational schools, junior colleges, and undergraduate programs. Vocational education primarily prepares practice-oriented nurses, junior colleges emphasize professional skill development, and undergraduate programs focus on integrating theoretical knowledge with clinical practice to cultivate comprehensive competencies (7).

Continuing education includes adult higher education, credit-based continuing education systems, and self-directed examination programs, which address the needs of in-service nurses for academic advancement and knowledge updating (6). At the postgraduate level, master’s and doctoral programs aim to cultivate advanced clinical and research-oriented nursing professionals, thereby strengthening research capacity and professional leadership within the discipline (6).

Although this multi-level training system produces a broad range of nursing professionals, it also creates challenges for the standardized development of ethical competence. Students at different educational levels vary considerably in their knowledge base, cognitive development, and learning capacity, making it difficult to implement a unified framework for ethics education. Existing instruments also have limitations. For example, Zhao and Zhuang (8) applied the Nursing Leaders’ Ethical Decision-Making Confidence Scale to assess nursing managers, Chen et al. (9) developed the Nursing Ethical Decision-Making Competence Assessment Scale, and Zhang (10) validated the Nursing Ethical Behavior Questionnaire. However, these tools are generally context-specific and may not be suitable for nursing students across diverse educational levels, nor do they comprehensively capture the process of ethical decision-making in practice.

Against this background, it is essential to assess ethical competence accurately among nursing students from different educational pathways. A clear understanding of their current competence levels can inform targeted improvements in ethics education and contribute to the development of a high-quality nursing workforce. In 2024, Pai and Hwu developed the Ethical Decision-Making Competence Scale (EDM-CS) on the basis of Rest’s Four-Component Model (11). The instrument provides a theoretically grounded and structured approach to evaluating ethical decision-making competence and has shown good psychometric properties in Taiwan, China (12).

However, because mainland China and Taiwan differ in social context, educational systems, and healthcare environments, the applicability of the EDM-CS in mainland China requires further verification. In particular, the diversity of nursing education pathways in mainland China suggests that students at different levels may show distinct patterns of ethical decision-making (6, 13). Therefore, the reliability and validity of the EDM-CS across multiple educational levels in mainland China remain to be established. The present study therefore aimed to translate, culturally adapt, and validate the EDM-CS among nursing students across different educational levels in mainland China through a multicenter design.

## Methods

This multicenter cross-sectional study was conducted to translate, culturally adapt, and psychometrically validate the Chinese version of the Ethical Decision-Making Competence Scale (EDM-CS) among nursing students in mainland China. Reliability and validity were examined through item analysis, internal consistency, split-half reliability, test–retest reliability, content validity, exploratory factor analysis, confirmatory factor analysis, convergent validity, and discriminant validity.

### Design and participants

Participants were recruited by convenience sampling from nursing schools and affiliated teaching hospitals in four provinces of China, namely Guizhou, Guangdong, Henan, and Hunan.

Eligible participants were nursing students enrolled either in on-campus theoretical training or in hospital-based internships during the study period. Students receiving theoretical instruction on campus were recruited through campus announcements and online learning platform notifications. Intern students were recruited synchronously from 30 tertiary hospitals in which they were undertaking clinical placements. Data collection was conducted from December 2024 to March 2025 following preliminary technical and ethical screening through the institutional review process in late 2024. Inclusion criteria were willingness to participate and current enrollment in nursing education. Exclusion criteria were sick leave, maternity leave, or a recent major psychological stress event within the previous month.

### Measures

#### General population characteristics questionnaire

Based on an extensive literature review and group discussion, nine demographic and educational variables that might be associated with ethical decision-making competence were included in the general questionnaire: gender, grade, educational attainment, plans for the coming year, hospital level, willingness to engage in nursing, whether the clinical instructor attached importance to nursing ethics, whether nursing ethics courses had been taken, and whether ethics-related training had been received.

#### Ethical decision-making competence scale (EDM-CS)

The Ethical Decision-Making Competence Scale was developed on the basis of Rest’s four core ethical competencies (11). It contains 25 items across four dimensions: Ethical Judgment, Ethical Sensitivity, Ethical Motivation, and Ethical Action. Each item is rated on a 5-point Likert scale ranging from 1 (strongly disagree) to 5 (strongly agree), with higher scores indicating stronger ethical decision-making competence. In the original study, the overall Cronbach’s alpha coefficient was 0.90, and the coefficients for the four subscales ranged from 0.73 to 0.80 (12).

### Procedures

#### Translation and cultural adaptation

Permission to translate and use the original EDM-CS was obtained from the scale developer by email before the study began. The translation and cross-cultural adaptation process followed standardized methodological procedures, including forward translation, synthesis, back-translation, expert review, and pilot testing (14–16). First, two bilingual English teachers independently translated the original English version into Chinese. Two senior nursing professors then compared and synthesized the two Chinese translations, with particular attention to conceptual equivalence, professional terminology, and semantic clarity. The reconciled Chinese version was then back-translated into English by two bilingual Filipino-Chinese nursing educators and one bilingual language specialist based in the Philippines, none of whom had been exposed to the original scale. The back-translators worked independently and were blinded to the original English wording during the translation stage. The research team then compared the back-translated version with the original instrument and resolved discrepancies through discussion. This process was followed by expert content validation to further confirm semantic, conceptual, and cultural equivalence.

To ensure conceptual and cultural equivalence, expert content validation was conducted during the cultural adaptation stage. Six experts were invited to evaluate each item in terms of semantic accuracy, cultural appropriateness, and practical applicability, including one professor of nursing management, two registered nurses with at least 10 years of clinical experience, and three nursing postgraduates specializing in nursing ethics. All experts used a 4-point relevance scale, in which ratings of 3 or 4 were considered content-valid for calculation of the item-level content validity index (I-CVI) and the scale-level content validity index (S-CVI). Based on expert feedback, ambiguous wording and culturally inappropriate expressions were revised to produce the pre-final Chinese version of the EDM-CS.

**Figure 1:**
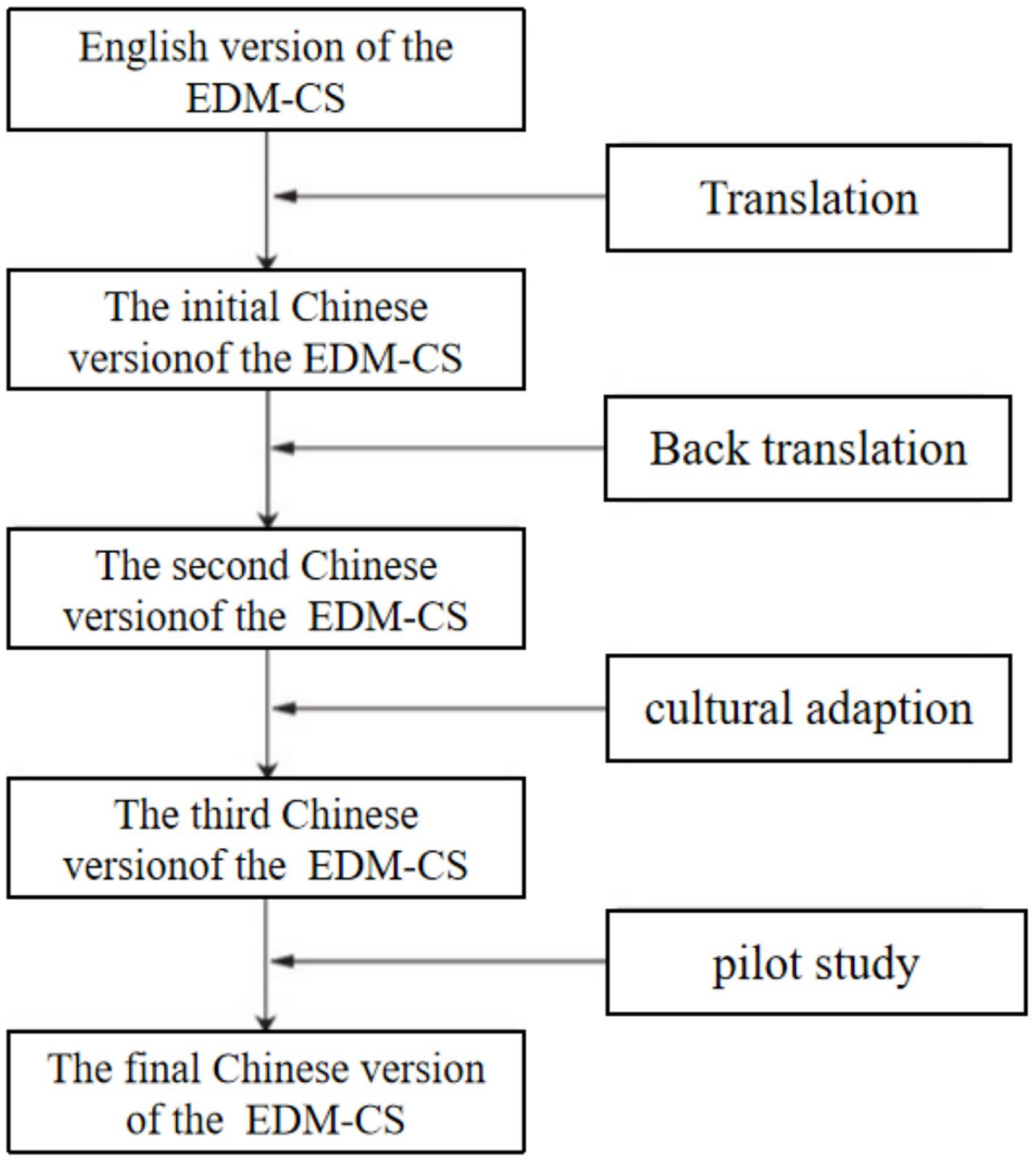
Translation and cross-cultural adaptation process of the EDM-CS.

A pilot test was then conducted with five nursing students to assess item clarity, comprehensibility, and the feasibility of questionnaire administration before the formal survey. The pilot participants reported that the questionnaire was easy to understand and could be completed within 3–5 min. Minor wording adjustments were made accordingly (Figure 1).

#### Data collection

Data were collected between December 2024 and March 2025. Before completing and submitting the questionnaire, all participants were presented with an online informed consent statement, study agreement, and privacy statement. Participants were required to read these materials before submission, and completion of the online questionnaire was taken as provision of informed consent.

Questionnaires were administered on site in schools and hospitals. A total of 1,409 questionnaires were collected. Because the Wenjuanxing platform was used, all items were set as mandatory to avoid missing data (17, 18). Two participants withdrew for personal reasons, and 1,407 valid questionnaires were ultimately included in the final analysis.

Content validity was evaluated by the same panel of six experts described above using the 4-point relevance scale. To assess test–retest reliability, a subsample of 140 participants completed the Chinese EDM-CS again 2 weeks after the initial survey. This subsample was selected from the original 1,407 participants using purposive quota-balanced sampling to ensure coverage of key demographic strata for stability assessment rather than to statistically mirror the full sample distribution. Specifically, a 50% gender quota was applied (70 males and 70 females). Within each gender group, participants were further recruited to achieve an educational distribution of 40% undergraduates (*n* = 28), 30% junior college students (*n* = 21), and 30% postgraduates (*n* = 21). Recruitment was conducted through a voluntary follow-up invitation, and each demographic cell remained open until the prespecified quota was reached. No additional proportional matching by placement status or province was imposed; however, all follow-up participants were drawn from the original multicenter cohort, which included students in both on-campus study and hospital-based placements across four provinces. Both EFA and CFA were conducted on the full sample of 1,407 participants.

#### Statistical analysis and psychometric evaluation

All statistical analyses were conducted using SPSS version 26.0 and SPSSAU (19, 20). Descriptive statistics, including means, standard deviations, frequencies, and percentages, were used to summarize participants’ demographic characteristics and item responses (21). Statistical significance was set at *p* < 0.05 (two-tailed).

#### Item analysis and reliability

Item analysis was performed to evaluate the discrimination and homogeneity of the scale items. Participants were divided into high-score and low-score groups on the basis of the 27th and 73rd percentiles of the total score, and independent-samples t-tests were conducted to calculate the critical ratio for each item (21, 22). Item-total correlation coefficients were computed to assess item homogeneity, with coefficients of at least 0.40 considered acceptable (19).

Internal consistency reliability was assessed using Cronbach’s alpha coefficients for the total scale and each subscale. Split-half reliability was calculated using the Spearman-Brown coefficient, and test–retest reliability was evaluated in the subsample that completed the questionnaire again after a two-week interval (23, 24). Reliability coefficients of at least 0.70 were considered acceptable (25).

#### Exploratory factor analysis

Exploratory factor analysis was conducted to examine the underlying factor structure of the Chinese EDM-CS. Before factor extraction, sampling adequacy and factorability were evaluated using the Kaiser-Meyer-Olkin measure and Bartlett’s test of sphericity. A KMO value of at least 0.60 and a significant Bartlett’s test were considered appropriate for factor analysis (20, 26). Because both EFA and CFA were performed on the full sample of 1,407 participants, the CFA findings should be interpreted as confirmation within the same dataset rather than as independent external validation.

Factors were extracted using principal component analysis, followed by orthogonal Varimax rotation to obtain a more interpretable structure (27, 28). Factors were retained on the basis of eigenvalues greater than 1.0 and inspection of the scree plot (22). Factor loadings of at least 0.40 were considered meaningful, and items with substantial cross-loadings were examined for potential removal (26).

#### Confirmatory factor analysis

Confirmatory factor analysis was conducted to examine the four-factor structure identified by the EFA. Given that the same full sample was used for both EFA and CFA, the CFA results were interpreted conservatively as evidence of structural coherence rather than independent replication. Convergent validity was evaluated using standardized factor loadings, average variance extracted (AVE), and composite reliability (CR), with AVE values of at least 0.50 and CR values of at least 0.70 indicating acceptable convergent validity (5, 12). Discriminant validity was further examined using the Heterotrait-Monotrait ratio.

### Ethical considerations

This study underwent a two-phase institutional ethical review procedure by the Medical Ethics Committee of Hunan University of Medicine. The study protocol first completed preliminary technical and ethical screening in late 2024, which authorized the initiation of data collection in December 2024. The formal approval was subsequently archived under Approval No. 2025(H03005). As this study was conducted using an online questionnaire survey, separately signed written informed consent was not required. Instead, informed consent information was presented on the first page of the online survey platform. Participants were informed of the purpose of the study, the voluntary nature of participation, and confidentiality protections before proceeding to the questionnaire.

## Results

### General information

A total of 1,407 participants were included in the study. The mean age was 20.30 ± 3.06 years, and the mean total EDM-CS score was 73.94 ± 16.21. Among the participants, 227 (16.13%) were men and 1,180 (83.87%) were women. Additional demographic characteristics are presented in Table 1.

**Table 1:** General demographic characteristics (*n* = 1,407).

Variable	Category	*n*	%
Grade	Freshman undergraduate	84	5.97
	Sophomore undergraduate	502	35.68
	Junior undergraduate	594	42.22
	Senior undergraduate	173	12.30
	Fifth-year undergraduate	6	0.43
	First-year postgraduate	20	1.42
	Second-year postgraduate	8	0.57
	Third-year postgraduate	5	0.36
	Other	15	1.07
Gender	Male	227	16.13
	Female	1,180	83.87
Educational attainment	Undergraduate	1,143	81.24
	Junior college	226	16.06
	Postgraduate	38	2.70
Hospital grade	Third-class Grade A	471	33.48
	Third-class Grade B	42	2.99
	Second-class Grade A	18	1.28
	Studying at school	876	62.26
Plans for the coming year	Clinical nursing	853	60.63
	Pursue a master’s degree	247	17.56
	Nursing teacher	80	5.69
	Switch careers	227	16.13
Willingness to engage in nursing	Unwilling	288	20.47
	Willing	557	39.59
	I do not care	562	39.94
Instructor attaches importance to nursing ethics	Yes (received in hospital)	704	50.04
	No (not received in hospital)	44	3.13
	Not yet exposed (currently in school)	659	46.84
Studied nursing ethics courses	Yes	1,080	76.76
	No	327	23.24
Received ethics-related training	Yes	839	59.63
	No	568	40.37

### Item analysis

The critical ratios ranged from 19.374 to 29.776, and the item-total correlation coefficients ranged from 0.536 to 0.749. The Cronbach’s alpha coefficient if item deleted ranged from 0.943 to 0.946, indicating that no item removal would improve the internal consistency of the scale. Detailed results are shown in Table 2.

**Table 2:** Item analysis for the Chinese version of the EDM-CS.

Dimension	Item	CR	Item-total *r*	Alpha if deleted
Ethical Judgment	1. I can analyze the obstacles that patients and their families encounter when making decisions.	20.060**	0.536**	0.946
	2. I can recognize the obstacles that patients and their families face in decision-making.	28.402**	0.641**	0.945
	3. I can recognize the hidden risks and benefits within an ethical dilemma.	26.684**	0.625**	0.945
	4. I can determine the ethical appropriateness of caring for patients with complex conditions.	25.471**	0.624**	0.945
	5. I can identify and manage my own and others’ moral distress.	26.639**	0.628**	0.945
	6. I can identify genuine ethical dilemmas in practice.	19.374**	0.595**	0.945
	7. I can identify conflicts between patients and their families during decision-making.	20.600**	0.596**	0.945
	8. I can make sound ethical decisions.	20.034**	0.598**	0.945
Ethical Sensitivity	9. I can hear the decisions being made by patients and their families.	25.076**	0.687**	0.944
	10. I am willing to listen to how patients and their families feel regarding decision-making dilemmas.	24.693**	0.662**	0.944
	11. I can encourage patients and their families to express the decision-making issues they face.	26.662**	0.705**	0.944
	12. I actively care for and assist patients and their families with decision-making issues.	25.760**	0.682**	0.944
	13. I can maintain a good relationship with patients and their families when decision-making problems arise.	24.232**	0.677**	0.944
	14. I understand the difficulties and conflicts involved in decision-making between patients and their families.	28.413**	0.719**	0.944
	15. I can consult with teams to assist in ethical decision-making dilemmas.	23.446**	0.695**	0.944
	16. I can understand and guide the feelings of patients and their families during decision-making dilemmas.	22.278**	0.702**	0.944
Ethical Motivation	17. I can cultivate and lead others to uphold ethical practice in my work.	22.052**	0.703**	0.944
	18. I can explain my ethical decisions using appropriate ethical terms and language.	19.630**	0.677**	0.944
	19. I can engage in preventive and ethical initiatives to address ethical issues in my field.	19.415**	0.582**	0.945
	20. I can explain the ethics of care based on my professional expertise.	20.683**	0.661**	0.944
	21. I can plan ethical priorities in practice.	23.819**	0.709**	0.944
Ethical Action	22. I can communicate the most appropriate treatment to patients and their families clearly and understandably.	29.776**	0.749**	0.943
	23. I can explain health-care issues to patients and their families in plain and comprehensible language.	27.093**	0.719**	0.944
	24. I can apply ethical principles in a timely manner to inform care-related decision-making by patients and families.	27.125**	0.727**	0.943
	25. I can understand ethical issues and provide ethical advice to patients and their families.	20.658**	0.636**	0.945

**Indicates *p* < 0.01.

### Reliability analysis

The overall Cronbach’s alpha coefficient of the Chinese EDM-CS was 0.946. The alpha coefficients of the four subscales ranged from 0.869 to 0.904. Split-half reliability for the total scale was 0.933. For test–retest reliability, the quota-sampled subsample of 140 participants completed the scale again 2 weeks later, yielding a coefficient of 0.856. These findings indicate satisfactory reliability (30) (Table 3).

**Table 3:** Reliability analysis for the Chinese version of the EDM-CS.

Scale or dimension	Cronbach’s alpha	Split-half reliability	Test–retest reliability
EDM-CS	0.946	0.933	0.856
Ethical Judgment	0.891	—	—
Ethical Sensitivity	0.896	—	—
Ethical Motivation	0.869	—	—
Ethical Action	0.904	—	—

### Validity analysis

#### Exploratory factor analysis

**Figure 2:**
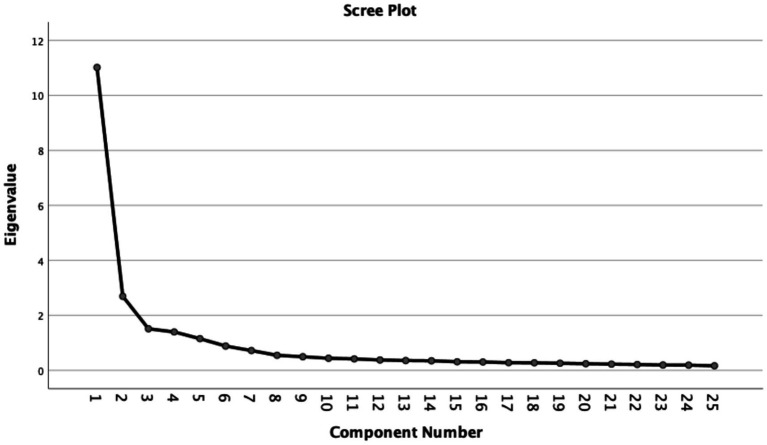
Scree plot of the exploratory factor analysis for the Chinese version of the EDM-CS.

The Kaiser-Meyer-Olkin value was 0.942, and Bartlett’s test of sphericity was significant (chi-square = 25,297.281, *p* < 0.001), indicating that the data were suitable for factor analysis. Four factors were extracted, accounting for 71.07% of the total variance. The scree plot also supported retention of four factors (Figure 2). Factor loadings ranged from 0.730 to 0.846, and no substantial cross-loadings were observed.

#### Content validity analysis

Six experts evaluated the relevance of the translated items. The item-level content validity index ranged from 0.833 to 1.000, and the scale-level content validity index was 0.920, indicating satisfactory content validity of the Chinese version of the EDM-CS (29).

#### Confirmatory factor analysis

**Figure 3:**
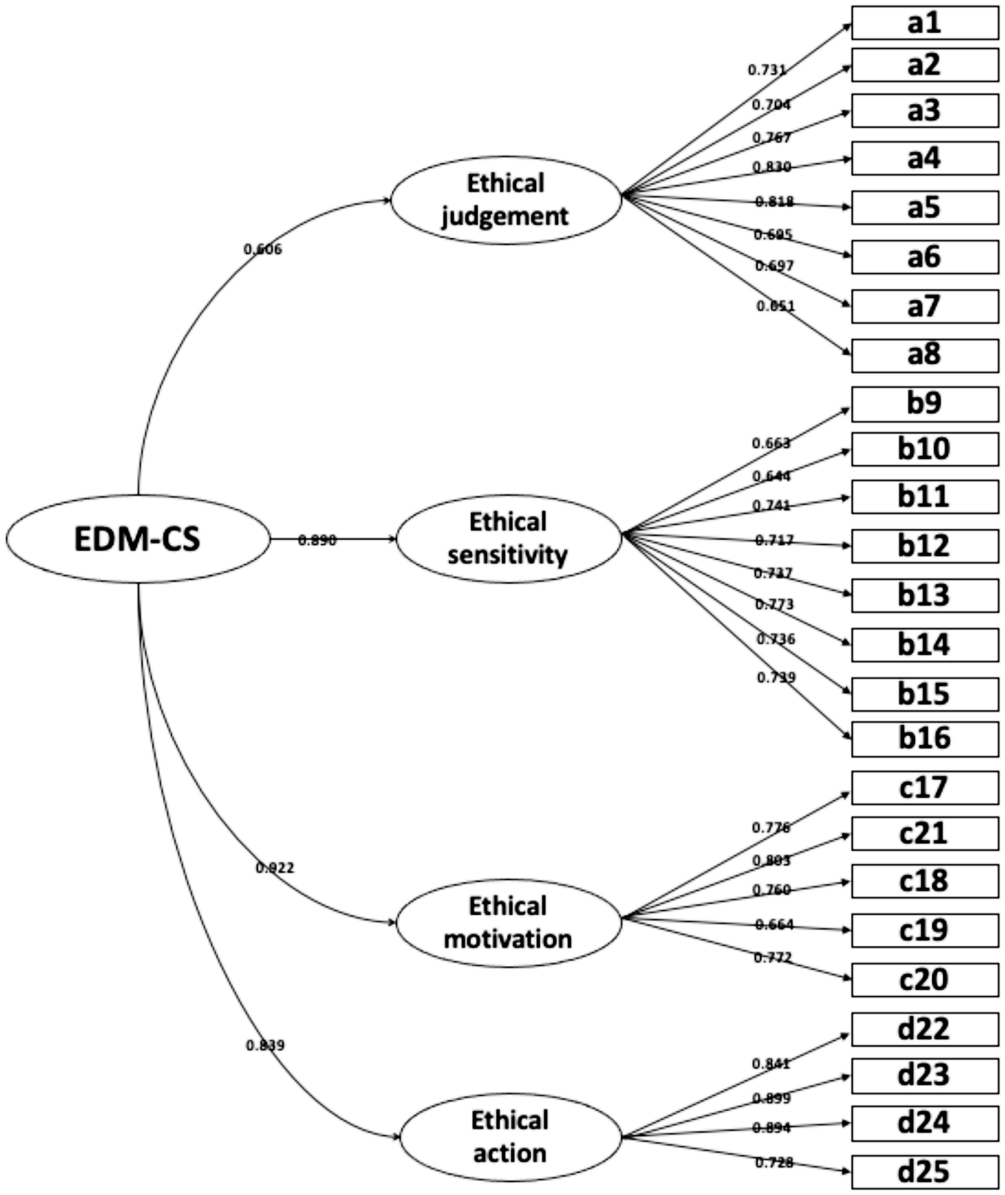
Standardized four-factor model of the Chinese version of the EDM-CS.

Confirmatory factor analysis was performed for the four-factor, 25-item model. The standardized four-factor structure is shown in Figure 3. AVE values for the four dimensions ranged from 0.518 to 0.711, and CR values ranged from 0.870 to 0.907, indicating adequate convergent validity (Table 4). To provide a more sensitive examination of discriminant validity, HTMT ratios were also calculated. As shown in Table 5, all HTMT ratios ranged from 0.486 to 0.818, which were below the conservative threshold of 0.85 and therefore supported adequate discriminant validity among the four dimensions.

**Table 4:** Convergent validity and inter-factor correlations of the Chinese EDM-CS.

Factor	Ethical Judgment	Ethical Sensitivity	Ethical Motivation	Ethical Action	AVE	CR
Ethical Judgment	0.740				0.548	0.894
Ethical Sensitivity	0.603	0.720			0.518	0.896
Ethical Motivation	0.429	0.713	0.757		0.572	0.870
Ethical Action	0.472	0.646	0.726	0.843	0.711	0.907

Diagonal values represent the square roots of the AVE. AVE, average variance extracted; CR, composite reliability.

**Table 5:** HTMT ratios for discriminant validity.

Factor	Factor 1	Factor 2	Factor 3	Factor 4
Factor 1	—			
Factor 2	0.673	—		
Factor 3	0.486	0.810	—	
Factor 4	0.526	0.718	0.818	—

### Dimension-theory mapping

The four factors extracted by EFA and supported by CFA were explicitly mapped onto Rest’s Four-Component Model of ethical decision-making (11). Ethical Judgment corresponded to moral judgment, reflecting the ability to analyze ethical dilemmas, identify risks and benefits, and make sound ethical decisions. Ethical Sensitivity corresponded to moral sensitivity, reflecting the ability to recognize ethical dilemmas in clinical practice and perceive the needs of patients and their families. Ethical Motivation corresponded to moral motivation, reflecting the willingness to prioritize ethical principles in practice, guide others to uphold ethics, and plan ethical priorities. Ethical Action corresponded to moral action, reflecting the ability to communicate ethical decisions clearly to patients and families and translate ethical judgments into practical care behaviors.

This mapping indicates that the Chinese version of the EDM-CS retains the theoretical foundation of the original scale and remains aligned with a widely accepted framework of ethical decision-making in nursing research (2, 12).

## Discussion

### Principal findings

This study systematically translated and validated the Chinese version of the Ethical Decision-Making Competence Scale among nursing students across multiple educational levels in mainland China (33). The findings demonstrate that the Chinese EDM-CS has robust psychometric properties, including excellent internal consistency (Cronbach’s alpha = 0.946), satisfactory test–retest reliability (0.856), and sound construct validity. The original four-factor structure was retained, explaining 71.07% of the total variance. In addition, convergent and discriminant validity were supported by the AVE, CR, and HTMT results, suggesting that the scale is both structurally coherent and theoretically meaningful (31, 34, 36).

### Comparison with previous studies

The present findings are consistent with the original validation study conducted by Pai and Hwu (12), in which the EDM-CS showed good reliability and validity among nursing populations in Taiwan. The internal consistency observed in the present study was slightly higher than that reported in the original version, which may be related to the large and heterogeneous multicenter sample drawn from several educational levels in mainland China.

Compared with existing instruments developed in mainland China, such as the Nursing Ethical Decision-Making Competence Assessment Scale and the Ethical Behavior Questionnaire, the EDM-CS provides a more comprehensive and theoretically grounded framework. Rather than focusing on a single professional group or a narrow clinical context, the EDM-CS captures the process of ethical decision-making across Ethical Sensitivity, Ethical Judgment, Ethical Motivation, and Ethical Action (32). It therefore helps address an important gap in the assessment of nursing students from diverse educational pathways.

### Theoretical implications

A major strength of this study is the explicit alignment between the empirical factor structure and Rest’s Four-Component Model of ethical decision-making (4–6, 11, 12). The four extracted dimensions closely correspond to moral sensitivity, moral judgment, moral motivation, and moral action, respectively. This correspondence is not merely a *post hoc* labeling exercise; rather, it provides empirical support for the applicability of Rest’s model within the context of mainland Chinese nursing education.

Notably, the correlation between Ethical Motivation and Ethical Action was relatively high (*r* = 0.726). Within Rest’s framework, these two components are sequentially linked and therefore expected to be related, although they remain conceptually distinct. The strong correlation observed here may reflect both contextual and theoretical considerations. From a contextual perspective, nursing practice in mainland China often takes place in highly standardized clinical environments, where institutional protocols, professional norms, and workflow routines may facilitate a relatively direct translation of moral motivation into action. From a theoretical perspective, this does not necessarily contradict Rest’s sequential model. Rather, it may indicate that adjacent components of moral functioning, while conceptually distinguishable, can become more tightly coupled in practice under conditions where role expectations and action pathways are clearly structured. Nevertheless, the two constructs remained distinguishable in the present study, as the corresponding HTMT value was 0.818, which remained below the conservative threshold of 0.85.

At the same time, because both EFA and CFA were conducted on the same full sample, the CFA findings should be interpreted cautiously. The present results support structural coherence within the current dataset, but further confirmation in an independent sample is still needed. Even so, the present findings suggest that ethical decision-making competence among nursing students can be conceptualized as a multidimensional and sequential process, thereby reinforcing the theoretical relevance of the EDM-CS in mainland Chinese nursing education.

### Practical implications

From a practical perspective, the validated Chinese version of the EDM-CS provides a reliable and standardized instrument for assessing ethical decision-making competence among nursing students at different educational levels. This has important implications for nursing education, curriculum development, and competency-based training.

Specifically, educators can use the scale to identify weaknesses in particular dimensions of ethical competence and design targeted interventions accordingly. The instrument may also support the evaluation of ethics education programs and contribute to the development of more standardized training frameworks across institutions. At a broader level, the use of this tool may facilitate improvements in ethical practice in clinical settings and ultimately enhance the quality of patient care.

### Strengths and limitations

Several limitations should also be acknowledged. Convenience sampling may have introduced selection bias and may limit representativeness. The study also relied on self-reported data, which raises the possibility of response bias. In addition, the proportion of postgraduate participants was relatively small, which may limit subgroup-level interpretation. Finally, because EFA and CFA were conducted on the same dataset, independent replication remains necessary. Furthermore, the present cross-sectional validation design does not allow conclusions about temporal or causal ordering between the identified components. Future longitudinal studies are therefore required to further examine the relationship between moral motivation and action, thereby addressing the methodological limits of the present research.

## Conclusion

The Chinese version of the EDM-CS demonstrates good reliability and validity and is suitable for assessing ethical decision-making competence among nursing students in mainland China. The scale helps address the lack of standardized assessment instruments in this area and contributes to the broader literature on nursing ethics education (35). Future research should further examine its performance in independent samples, its longitudinal application, and its usefulness for evaluating educational interventions.

## Data Availability

The raw data supporting the conclusions of this article will be made available by the authors, without undue reservation.
